# Rat intestinal mast cell amines are released during nitric oxide synthase inhibition *in vitro*

**DOI:** 10.1155/S0962935196000051

**Published:** 1996-02

**Authors:** A. M. Northover, B. J. Northover

**Affiliations:** 1 Department of Pharmaceutical Sciences School of Applied Sciences De Montfort University Leicester LE1 9BH UK

## Abstract

Inhibition of nitric oxide synthase increases microvascular permeability in rat small intestinal villi. To determine the mechanism(s) whereby this occurs we have perfused the vasculature of rat isolated small intestines with a gelatin-containing physiological salt solution. Inclusion of N-nitro-L-argintne methyl ester (L-NAME, 100 μM) or indomethacin (1 μM) in the perfusate increased leakage of injected colloidal carbon into microvessel walls. Pre-treatment with sodium nitroprusside (10 μM) significantly reduced the effects of both L-NAME and indomethacin, whereas carbacyclin (1 μM) only reduced the effects of indomethacin. PD151242 (1 μM) showed some antagonism towards the effects of L-NAME, but nordihydroguaiaretic acid (3 μM) was inactive. Pre-tment with cyproheptadine (10 μM) reduced the effects of both L-NAME and indomethacin, and also significantly reduced background (control) colloidal carbon leakage. Small intestines from polymixin B-treated rats showed significantly reduced colloidal carbon leakage in response to L-NAME. This suggests that the leakage-enhancing effects of both L-NAME and indomethacin in this preparation may be mediated by mast cell-derived amines.


Mediators of Inflammation 5, 32–36 (1996)

### Rat intestinal mast cell amines are released during nitric oxide synthase inhibition *in vitro*

A. M. Northover[CA] and B. J. Northover

Department of Pharmaceutical Sciences, School of Applied Sciences, De Montfort University, Leicester, LE1 9BH, UK. Fax: (+44) (0)116 2577287.

[CA]Corresponding Author

**INHIBITION of nitric oxide synthase increases microvascular permeability in rat small intestinal villi. To determine the mechanism(s) whereby this occurs we have perfused the vasculature of rat isolated small intestines with a gelatin-containing physiological salt solution. Inclusion of *N*-nitro-L-arginine methyl ester (L-NAME, 100 μM) or indomethacin (1 μM) in the perfusate increased leakage of injected colloidal carbon into microvessel walls. Pre-treatment with sodium nitroprusside (10 μM) significantly reduced the effects of both L-NAME and indomethacin, whereas carbacyclin (1 μM) only reduced the effects of indomethacin. PD151242 (1 μM) showed some antagonism towards the effects of L-NAME, but nordihydroguaiaretic acid (3 μM) was inactive. Pre-treatment with cyproheptadine (10 μM) reduced the effects of both L-NAME and indomethacin, and also significantly reduced background (control) colloidal carbon leakage. Small intestines from polymixin B-treated rats showed significantly reduced colloidal carbon leakage in response to L-NAME. This suggests that the leakage-enhancing effects of both L-NAME and indomethacin in this preparation may be mediated by mast cell-derived amines.**

**Key words:** Carbacyclin, Colloidal carbon, Cyproheptadine, Indomethacin, Mast cell amines, Microvascular permeability, *N*-Nitro-L-arginine methyl ester, Nitric oxide, Nordihydroguaiaretic acid, PD151242, Polymixin B, Sodium nitroprusside

### Introduction

Injured tissues release a complex mixture of pro- and anti-inflammatory compounds. Several authors have shown recently, for example, that endogenous nitric oxide (NO) can protect rat small intestine/mesentery preparations that are injured, resulting in less increase in microvascular permeability to either albumin[1–5] or colloidal carbon,[6] or net fluid secretion into the intestinal lumen.[7] Microvascular leakage is generally thought to occur because of a loss of endothelial integrity, resulting from endothelial cell contraction.[8] Thus, endothelial cell relaxation in response to NO would be expected to bring about a recovery of this integrity. Therefore, just as vascular smooth muscles are known to relax in response to NO,[9] as a result of activation of guanylyl cyclase,[10] so too might endothelial cells.[6]

Prostacyclin and certain other prostanoids can also bring about vascular relaxation,[9] and their release from endothelial cells concomitantly with NO has been demonstrated to occur in response to inflammatory stimuli *in vivo*,[11–13] in isolated vessels[14,15] and in cultured cells.[16–18] Endothelin may also be released concomitantly with NO under these circumstances.[17,19] In addition, it has been suggested that normality in the intestinal vasculature is maintained by a balance between the opposing effects of secreted NO and various 5-lipoxygenase products.[20]

Ischaemia–reperfusion injury in rat small intestine causes a degranulation of mucosal mast cells.[2,21] Furthermore, mast cells release an NO-like factor which can in turn modulate the release of pro-inflammatory mediators.[22] Participation of mast cells in other forms of intestinal injury, however, is poorly understood.

In the present work we have attempted to correlate these various findings and suggestions using a rat isolated perfused small intestine/mesentery preparation, with injected colloidal carbon acting as a marker of increased microvascular permeability.

### Materials and Methods

*Preparation of small intestine:* The basic method has been described in detail previously.[23] Briefly, rats weighing 350–500 g were killed by inhalation

© 1996 Rapid Science Publishers of chloroform vapour. Immediately thereafter, a cannula was placed in the anterior mesenteric artery. The small intestine, with its accompanying blood vessels, was then ligated at its caecal and pyloric ends. Along with its mesentery, the intestine was next removed to an isolated-organ bath containing perfusate. The cannula was then connected to a reservoir of the same perfusate maintained at 37°C.

*Perfusion protocols:* With perfusate flowing at 10 ml/min the vasculature was first cleared of residual blood. The perfusate, with test drugs added as appropriate, was then re-circulated through the tissue for 17 min. Then 0.5 ml colloidal carbon suspension was introduced into the perfusion line near the cannula. The perfusate was thereafter allowed to run to waste for 8 min, using fresh perfusate for the last 6 min, and increasing the flow rate to 20 ml/min for the final 2 min to ensure removal of the last traces of colloidal carbon from the vessel lumens. Finally, 2 ml rat washed red blood cells were introduced near the cannula to provide a visual check for patency of the microvessels when the tissue was examined microscopically at the end of the experiment.

*Image analysis:* The small intestine was next removed from the organ bath to a tray containing normal saline. Starting at the caecal end, six segments, each approximately 4 cm in length, were cut. Each segment was flushed with normal saline, opened lengthwise along its mesenteric border and stapled to a xylene-resistant plastic coverslip. After fixation, dehydration, and a period of clearance in xylene, the specimens were mounted, mucosal surface uppermost, in DPX mountant on glass slides. Micrographs of villous microvessels in five widely separated areas of each specimen were taken at × 100 magnification. Negative micrographs were subjected to image analysis, and areas depicting colloidal carbon deposits in each field were expressed as a percentage of the frame (negative micrograph) area, as described earlier.[23]

*Perfusates:* The basic perfusate used was a physiological salt solution containing gelatin and had the following composition (mM): NaCl, 138; KCl, 5; $NaHCO_3$, 10.1; $MgCl_2$, 1.06; $NaH_2PO_4$, 0.416; $CaCl_2$, 2; glucose, 10; plus 2% gelatin, giving pH 7.4. It was gassed throughout with 95% $O_2$ and 5% $CO_2$ in both the organ bath and the reservoir.

In the first series of experiments those drugs to be tested as possible antagonists of colloidal carbon leakage were added to 50 ml of perfusate at the beginning of the 17 min re-circulation period, followed 2 min later, where appropriate, by addition of drugs that were being tested as possible promoters of colloidal carbon leakage.

In a second series of experiments those drugs being tested as possible antagonists were added to the perfusate from the outset, and thus were present even while flushing blood from the vasculature. Drugs being tested as agonists, however, were added only for the 15 min re-circulation period.

In a third series of experiments rats were injected i.p. with a single dose of 10 000 units of polymixin B 3 days prior to being used to provide tissue for *in vitro* perfusion. Polymixin B at this dose was reported to deplete rat mast cells of stored amines.[24]

*Chemicals:* *N*-nitro-L-arginine methyl ester (L-NAME, 100 μM) and indomethacin (1 μM) were tested as possible agonists. Sodium nitroprusside (10 μM), carbacyclin (1 μM), cyproheptadine HCl (10 μM), nordihydroguaiaretic acid (3 μM) and PD151252 (1 μM) were tested as possible antagonists. L-NAME, indomethacin, sodium nitroprusside, cyproheptadine, PD151242 and polymixin B were used as aqueous solutions. Carbacyclin was dissolved first in ethanol, and nordihydroguaiaretic acid in dimethylsulphoxide. A dose–volume of 0.2 ml was added to 50 ml of perfusate in the reservoir.

L-NAME, carbacyclin, nordihydroguaiaretic acid and polymixin B were purchased from Sigma Chemical Co. (Poole, UK); sodium nitroprusside from David Bull Laboratories (Warwick, UK); indomethacin and cyproheptadine from Merck, Sharpe & Dohme Ltd (Hoddesdon, UK). PD151242 was a gift from Dr A. Doherty, Parke Davis (Ann Arbor, MI, USA). Gelatin and DPX mountant were obtained from BDH (Poole, UK); Thermanox® xylene-resistant coverslips from Nunc Inc. (Naperville, IL, USA) and colloidal carbon (Gunther Wagner, Batch C11/1431a) from Pelikan Inks (Hanover, Germany).

*Statistics:* Bonferroni's test was used for comparing multiple groups with a single control group.

### Results

*Effects of L-NAME and indomethacin on colloidal carbon leakage:* L-NAME (100 μM), an NO synthase inhibitor,[25] increased the leakage of colloidal carbon into villous microvessel walls (Table 1), confirming earlier work.[6] Indomethacin (1 μM), a cyclooxygenase inhibitor,[26] also increased colloidal carbon leakage (Table 1).

Table 1. Effects on colloidal carbon leakage in rat small intestinal villous microvessels of adding L-NAME or indomethacin (Indo) to the perfusate for 15 min after pre-treatment with various agents for 2 min

| Treatment | Amount of CC leakage (as % of frame area[a])[b] | | | | | |
|---|---|---|---|---|---|---|
| | *n* | GPSS | *n* | GPSS + L-NAME | *n* | GPSS + Indo |
| Control | 17 | $0.99 \pm 0.12^{c}$ | 10 | $2.54 \pm 0.30^{cc}$ | 10 | $2.19 \pm 0.23^{cc,d}$ |
| SNP | | ND | | ND | 10 | $1.37 \pm 0.19^{dd}$ |
| Carbacyclin | 4 | $0.91 \pm 0.22^{e}$ | 6 | $2.30 \pm 0.31^{ee}$ | 6 | $1.10 \pm 0.24^{dd}$ |
| NDGA | 4 | $1.28 \pm 0.23$ | 6 | $2.04 \pm 0.41$ | | ND |
| PD151242 | | ND | 6 | $1.70 \pm 0.23$ | | ND |
| Cypro | 6 | $0.32 \pm 0.08^{cc}$ | | ND | | ND |

[a]Each frame represents one negative micrograph magnified approximately 20 times. [b]Values are given as mean ± S.E.M. There are significant differences ($p < 0.05$, Bonferroni's test) between groups marked [c] and [cc]; between groups marked [d] and [dd]; and between groups marked [e] and [ee]. ND = Not done.

Abbreviations not used in text: GPSS = gelatin-containing physiological salt solution; SNP = sodium nitroprusside; NDGA = nordihydroguaiaretic acid; Cypro = cyproheptadine. Concentrations of L-NAME, indomethacin, SNP, carbacyclin, NDGA, PD151242 and cyproheptadine are given in the Methods section.

*Effects of adding sodium nitroprusside, carbacyclin, PD151242, nordihydroguaiaretic acid or cyproheptadine to the perfusate 2 min prior to L-NAME or indomethacin:* Sodium nitroprusside (10 μM), an NO donor,[27] has been shown earlier to significantly reduce the L-NAME-induced leakage of colloidal carbon in this preparation.[6] In the present work it also reduced the indomethacin-induced colloidal carbon leakage (Table 1). In contrast, pre-treatment with carbacyclin (1 μM), a less rapidly metabolized, synthetic analogue of prostacyclin,[28] significantly reduced the indomethacin-induced colloidal carbon leakage but had no effect on the leakage-response to L-NAME (Table 1). PD151242 (1 μM), an endothelin$_A$-receptor antagonist,[29] showed a trend towards reducing the L-NAME-induced colloidal carbon leakage, but nordihydroguaiaretic acid (3 μM), a 5-lipoxygenase inhibitor,[30] had no discernible effect (Table 1). None of the above named putative antagonists had any effect on the control (background) colloidal carbon leakage (Table 1). In contrast, addition to the perfusate of cyproheptadine (10 μM), a mixed histamine and 5-hydroxytryptamine receptor antagonist,[31] significantly reduced control (background) colloidal carbon leakage (Table 1).

*Effects of using perfusate containing sodium nitroprusside, carbacyclin or cyproheptadine throughout on the responses to L-NAME or indomethacin:* In agreement with the foregoing results, the continuous presence of cyproheptadine also significantly reduced control (background) colloidal carbon leakage (Table 2), whereas the continuous presence of sodium nitroprusside and carbacyclin, although tending now to reduce the background leakage of colloidal carbon, still failed to show a statistically significant effect ($p < 0.175$, Bonferroni's test for carbacyclin, whereas $p < 0.025$ Student *t*-test) (Table 2). Cyproheptadine also completely prevented both the L-NAME- and the indomethacin-induced leakage of colloidal carbon, reducing them to the same low value that was seen with cyproheptadine alone (Table 2).

*Effects of pre-treatment with polymixin B:* There was a significant reduction in colloidal carbon leakage in the L-NAME-treated group of preparations from rats pre-treated with polymixin B when compared with rats not so pre-treated (Table 2). However, there was no significant difference between rats pre-treated with polymixin B and those in the control group (Table 2).

### Discussion

In the present experiments both L-NAME and indomethacin significantly increased the leakage of colloidal carbon over and above control (background) values in rat villous microvessels (Table 1), suggesting that the release of both NO and a cyclooxygenase product contributed to endothelial integrity under these perfusion conditions. This interpretation is supported by the fact that pre-treatment with sodium nitroprusside reduced the leakage of colloidal carbon caused by both L-NAME and indomethacin. Carbacyclin, on the other hand, overcame the leakage-promoting effect of indomethacin but not that of L-NAME (Table 1). This suggests that the protective effects of exogenous carbacyclin, and hence probably of endogenous prostacyclin-like substances, occurred indirectly by increasing the availability of NO in the intestinal mucosa. They would not be expected to exert an effect, therefore, in the absence of NO synthase activity resulting from the presence of L-NAME. Interactions between the NO-synthase and cyclooxygenase pathways, however, seem to vary in different parts of the body. Thus, in some tissues

NO can release prostacyclin rather than vice versa. It has been shown, for example,[32,33] that the cyclooxygenase activity of macrophages *in vitro* can be increased either by enhancement of NO synthase activity or by treatment with an NO donor such as sodium nitroprusside. In contrast, exogenously applied prostacyclin was shown to release NO in pig pial arteries *in vivo*.[34] Reasons for these tissue differences remain to be explored.

5-Lipoxygenase is another enzyme that is involved in arachidonic acid metabolism, but it did not appear to be involved in the present experimental situation, since nordihydroguaiaretic acid failed to reduce the leakage-producing effects of L-NAME (Table 1). This contrasts with the report that BW A137C, another 5-lipoxygenase inhibitor,[30] can protect rat colonic microvessels against the permeability-enhancing effects of L-NAME.[20] However, the latter experiments were carried out *in vivo*, and with lipopolysaccharide co-administered to enhance the effects of L-NAME. In contrast, the present experiments were performed *in vitro*, and without augmentation of the actions of L-NAME. Which of these several experimental differences is responsible for the differing results obtained is not known, but it cautions against any simplistic extrapolation of the present findings to *in vivo* situations.

Rats injected i.v. with L-NAME have been reported to show modest increases in the plasma levels of endothelin-1.[35] Similarly, in the present experiments there was no significant reduction (Bonferroni's test) in the L-NAME-induced colloidal carbon leakage *in vitro* after pre-treatment with PD151242 (Table 1), although the reduction showed some significance after applying a Student *t*-test ($p < 0.05$). We have reported previously that exogenously applied endothelin-1 increases collodial carbon leakage in this preparation to some extent, but it is much more potent as a vasoconstrictor.[36]

Mast cell degranulation is known to be involved in ischaemia–reperfusion injury in the rat small intestine.[2,21] Moreover, Table 1 shows a reduction in background colloidal carbon leakage as a result of pre-treatment with cyproheptadine. Furthermore, Table 2 shows that colloidal carbon leakage is reduced to the same very low level by treatment with cyproheptadine both in the absence and presence of either L-NAME or indomethacin. This suggests that mast cell-derived histamine and/or 5-hydroxytryptamine mediate part of the colloidal carbon leakage seen in the controls, as well as the increased leakage shown by tissues exposed to L-NAME or indomethacin. It is particularly significant, therefore, that the small intestine/mesentery preparations derived from rats pre-treated with polymixin B, in order to deplete the mast cell stores of amines,[24,31] also showed very little colloidal carbon leakage in response to L-NAME. All these results point to mast cell-derived amines controlling microvascular permeability in this preparation, and it may be that these amines perform the same function as lipopolysaccharide *in vivo*.[3,4] Mast cell-derived mediators have been shown previously to be involved in the L-NAME-induced epithelial permeability enhancement seen in rat small intestine *in vivo*.[37] Since mast cells are also thought to secrete NO, and since this NO may actually stabilize those same cells which manufacture it,[22] experiments were performed in which sodium nitroprusside was added to the perfusate from the outset in order to mimic and augment the actions of endogenous NO. Background colloidal carbon leakage

**Table 2.** Effects on colloidal carbon leakage of perfusing rat small intestinal villous microvessels with gelatin-containing physiological salt solution (GPSS) containing various agents from the start of perfusion, or of pre-treating rats *in vivo* with polymixin B (PMX-B) 72 h prior to perfusion *in vitro*

| Perfusate | Treatment | *n* | Amount of CC leakage (as % of frame area[a])[b] |
|---|---|---|---|
| GPSS + Cypro | – | 7 | 0.28 ± 0.08[c] |
| GPSS + Cypro | L-NAME* | 5 | 0.29 ± 0.09[d] |
| GPSS + Cypro | Indomethacin* | 5 | 0.33 ± 0.12[e] |
| GPSS + SNP | – | 5 | 0.67 ± 0.04 |
| GPSS + Carbacyclin | – | 4 | 0.38 ± 0.10 |
| GPSS | PMX-B | 6 | 0.67 ± 0.12 |
| GPSS | PMX-B + L-NAME* | 5 | 0.89 ± 0.37[d] |

*L-NAME or indomethacin added for 15 min only. [a]Each frame represents one negative micrograph magnified 20 times. [b]Values are given as mean ± S.E.M. [c]$p < 0.05$ (Bonferroni's test) compared with GPSS control value in Table 1; [d]$p < 0.05$ (Bonferroni's test) compared with GPSS + L-NAME value in Table 1; [e]$p < 0.05$ (Bonferroni's test) compared with GPSS + Indo value in Table 1. Abbreviations not used in text: Cypro = cyproheptadine; SNP = sodium nitroprusside. Concentrations of Cypro, SNP, Carbacyclin, L-NAME and indomethacin are given in the Methods section.

was reduced by sodium nitroprusside, but not significantly so, suggesting that perhaps endogenous NO is more effective in this respect than the NO derived from sodium nitroprusside. If so, the greater effectiveness of carbacyclin over sodium nitroprusside on background colloidal carbon leakage may have been because carbacyclin acts by releasing endogenous NO, as suggested earlier.

The main conclusion to be drawn from the present work, therefore, is that some mast cell secretion occurs during perfusion of the rat intestinal vasculature with gelatin-containing physiological salt solution *in vitro*. Vascular leakage caused by this secretion is exacerbated by including either L-NAME or indomethacin in the perfusate, suggesting that NO and a prostanoid normally operate to restrain vascular permeability. It is uncertain whether this protection is due to prevention of the release of mast cell amines or an inhibition of their vascular effects.

### References

1. Hutcheson IR, Whittle BJR, Boughton-Smith NK. Role of nitric oxide in maintaining vascular integrity in endotoxin-induced acute intestinal damage in the rat. *Br J Pharmacol* 1990; **101:** 815–820.
2. Kurose I, Wolf R, Grisham MB, Granger DN. Modulation of ischemia/reperfusion-induced microvascular dysfunction by nitric oxide. *Circ Res* 1994; **74:** 376–382.
3. Laszlo F, Whittle BJR, Moncada S. Time-dependent enhancement or inhibition of endotoxin-induced vascular injury in rat intestine by nitric oxide synthase inhibitors. *Br J Pharmacol* 1994; **111:** 1309–1315.
4. László F, Whittle BJR, Moncada S. Interactions of constitutive nitric oxide with PAF and thromboxane on rat intestinal vascular integrity in acute endotoxaemia. *Br J Pharmacol* 1994; **113:** 1131–1136.
5. Liao L, Granger DN. Modulation of oxidized low-density lipoprotein-induced microvascular dysfunction by nitric oxide. *Am J Physiol* 1995; **268:** H1643–H1650.
6. Northover AM, Northover BJ. Rate of perfusion modulates colloidal carbon leakage from rat intestinal microvessels *in vitro*. *Mediators of Inflammation* 1995; **4:** 344–349.
7. Schirgi-Degen A, Beubler E. Significance of nitric oxide in the stimulation of intestinal fluid absorption in the rat jejunum *in vivo*. *Br J Pharmacol* 1995; **114:** 13–18.
8. Majno G, Shea SM, Leventhal M. Endothelial contraction induced by histamine-type mediators. An electron microscope study. *J Cell Biol* 1969; **42:** 647–672.
9. Furchgott RF. Role of endothelium in responses of vascular smooth muscle. *Circ Res* 1983; **53:** 557–573.
10. Moncada S, Palmer RMJ, Higgs EA. Nitric oxide; physiology, pathophysiology, and pharmacology. *Pharmacol Rev* 1991; **43:** 109–142.
11. Lopez-Belmonte J, Whittle BJR. The involvement of endothelial dysfunction, nitric oxide and prostanoids in the rat gastric microcirculatory responses to endothelin-1. *Br J Pharmacol* 1994; **112:** 267–271.
12. Tomlinson A, Appleton I, Moore AR, Gilroy DW, Willis D, Mitchell JA, Willoughby DA. Cyclo-oxygenase and nitric oxide synthase isoforms in rat carrageenin-induced pleurisy. *Br J Pharmacol* 1994; **113:** 693–698.
13. Sautebin L, Ialenti A, Ianaro A, Di Rosa M. Modulation by nitric oxide of prostaglandin biosynthesis in the rat. *Br J Pharmacol* 1995; **114:** 323–328.
14. De Nucci G, Thomas R, D'Orleans-Juste P, Antunes E, Walder C, Warner TD, Vane JR. Pressor effects of circulating endothelin are limited by its removal in the pulmonary circulation and by the release of prostacyclin and endothelium-derived relaxing factor. *Proc Natl Acad Sci USA* 1988; **85:** 9797–9800.
15. Mitchell JA, Chester AH, Borland JAA, Bishop-Bailey D, Larkin SW, Yacoub MH, Williams TJ. Co-induction of nitric oxide synthase and cyclo-oxygenase activity in human internal mammary artery. *Br J Pharmacol* 1995; **115:** 78P.
16. Lückhoff A, Pohl U, Mülsch A, Busse R. Differential role of extra- and intracellular calcium in the release of EDRF and prostacyclin from cultured endothelial cells. *Br J Pharmacol* 1988; **95:** 189–196.
17. D'Orléans-Juste P, Wood EG, Mitchell JA, Vane JR. Endothelin, EDRF and prostacyclin are released from bovine venous as well as arterial endothelial cells. *Br J Pharmacol* 1990; **99:** 100P.
18. Davidge ST, Baker PN, McLaughlin MK, Roberts JM. Nitric oxide produced by endothelial cells increases production of eicosanoids through activation of prostaglandin H synthase. *Circ Res* 1995; **77:** 274–283.
19. Durieu-Trautmann O, Fédérici C, Créminon C, *et al.* Nitric oxide and endothelin secretion by brain microvessel endothelial cells: regulation by cyclic nucleotides. *J Cell Physiol* 1993; **155:** 104–111.
20. László F, Whittle BJR. Colonic microvascular integrity in acute endotoxaemia: interactions between constitutive nitric oxide and 5-lipoxygenase products. *Eur J Pharmacol* 1995; **277:** R1–R3.
21. Boros M, Takaichi S, Masuda J, Newlands GFJ. Hatanaka K. Response of mucosal mast cells to intestinal ischemia–reperfusion injury in the rat. *Shock* 1995; **3:** 125–131.
22. Salvemini D, Masini E, Pistelli A, Mannaioni PF, Vane J. Nitric oxide: a regulatory mediator of mast cell reactivity. *J Cardiovasc Pharmacol* 1991; **17**(Suppl 3): S258–S264.
23. Northover AM. An *in vitro* method for assessing the effects of pro-inflammatory and anti-inflammatory compounds on microvascular permeability in the rat small intestine. *J Pharmacol Toxicol Meth* 1993; **29:** 227–232.
24. Levy L. Effects of different classes of drugs on the passive cutaneous anaphylaxis. *Arch Int Pharmacodyn* 1967; **165:** 92–102.
25. Rees DD, Palmer RMJ, Schulz R, Hodson HF, Moncada S. Characterization of three inhibitors of endothelial nitric oxide synthase *in vitro* and *in vivo*. *Br J Pharmacol* 1990; **101:** 746–752.
26. Vane JR. Inhibition of prostaglandin synthesis as a mechanism of action of aspirin-like drugs. *Nature (Lond)* 1971; **231:** 232–239.
27. Gruetter CA, Barry BK, McNamara DB, Gruetter DY, Kadowitz PJ, Ignarro LJ. Relaxation of bovine coronary artery and activation of coronary arterial guanylate cyclase by nitric oxide, nitroprusside and a carcinogenic nitrosoamine. *J Cyclic Nucleotide Res* 1979; **5:** 211–224.
28. Whittle BJR, Steel G, Boughton-Smith NK. Gastrointestinal actions of carbacyclin, a stable mimic of prostacyclin. *J Pharm Pharmacol* 1980; **32:** 603–604.
29. Davenport AP, Kuc RE, Fitzgerald F, Maguire JJ, Berryman K, Doherty AM. [$^{125}$I]-PD151242: a selective radioligand for human $ET_A$ receptors. *Br J Pharmacol* 1994; **111:** 4–6.
30. Tateson JE, Randall RW, Reynolds CH, Jackson WP, Bhattacherjee P, Salmon JA, Garland LG. Selective inhibition of arachidonate 5-lipoxygenase by novel acetohydroxamic acids: biochemical assessment *in vitro* and *ex vivo*. *Br J Pharmacol* 1988; **94:** 528–539.
31. Selye H, Somogyi A. Effect of cyproheptadine upon acute inflammation produced by various agents. *Med Pharm Exp* 1967; **17:** 255–263.
32. Salvemini D, Misko TP, Masferrer JL, Seibert K, Currie MG, Needleman P. Nitric oxide activates cyclooxygenase enzymes. *Proc Natl Acad Sci USA* 1993; **90:** 7240–7244.
33. Swierkosz TA, Mitchell JA, Warner TD, Botting RM, Vane JR. Co-induction of nitric oxide synthase and cyclo-oxygenase: interactions between nitric oxide and prostanoids. *Br J Pharmacol* 1995; **114:** 1335–1342.
34. Armstead WM. Role of nitric oxide and cAMP in prostglandin-induced pial artery vasodilation. *Am J Physiol* 1995; **268:** H1436–H1440.
35. Richard V, Hogie M, Clozel M, Löffler B-M, Thuillez C. *In vivo* evidence of an endothelin-induced vasopressor tone after inhibition of nitric oxide synthesis in rats. *Circulation* 1995; **91:** 771–775.
36. Northover AM, Northover BJ. Possible bi-directional link between $ET_A$ receptors and protein kinase C in rat blood vessels. *Mediators of Inflammation* 1995; **4:** 55–59.
37. Kanwar S, Wallace JL, Befus D, Kubes P. Nitric oxide synthesis inhibition increases epithelial permeability via mast cells. *Am J Physiol* 1994; **266:** G222–G229.

**Received 16 October 1995;**
**accepted in revised form 17 November 1995**
